# The Institutional Library

**Published:** 1920-09-11

**Authors:** 


### THE INSTITUTIONAL LIBRARY.

**The Nutrition of the Fœtus.** By J. MORRIS SLEMONS. (Oxford University Press; Humphrey Milford. Pp. 49. Price 3s. 6d. net.)

THE author claims to have shown experimentally that fœtal proteins are synthesised by the fœtus itself from animo-acids present in the maternal blood and diffused through the placenta into the fœtal circulation, as against the opposite view that the synthesis of fœtal proteins occurs in the placenta itself. The practical upshot is a recommendation which the best clinical opinion has long ago endorsed, that prospective mothers should not overeat themselves on protein foods. The paper in which this is expounded is reprinted from the *American Journal of Obstetrics*; it is a little difficult to understand why it should be thought necessary to reprint it as a book at a cost quite disproportionate to the importance of the thesis maintained in it.

**The Extra Pharmacopœia.** Vol. I., 17th edition. By MARTINDALE and WESTCOTT. Pages 1115. (Published by H. K. Lewis and Co., Ltd., Gower Street, London, W.C.)

THE appearance of the seventeenth edition of this well-known work is an obvious necessity arising firstly with the period of some four years since the publication of its predecessor and the remarkable changes which war's lessons have meanwhile taught us in the uses and manufactures of chemicals and drugs. A wholesale revision of the standing matter has been effected, and generally the authors have done their utmost to keep abreast of the extraordinarily rapid march of modern medical science.

**An Introduction to Midwifery.** By PROFESSOR A. DONALD, M.D. 8th edition. 1920. (C. Griffin and Co. Pp. 192. Price 6s.)

PROFESSOR DONALD'S condensation into the limits of a small textbook of such practical knowledge of obstetrics as a midwife or midwifery nurse may be expected to display is a fine piece of work. The new edition fully maintains the reputation of the former ones, and can be confidently recommended to midwifery students as one of the best elementary introductions to that subject that they are likely to come across.

**Manual of Hygiene for Students.** By JOHN GLAISTER, M.D., D.P.H. (Camb.). Third edition. (Edinburgh: E. and S. Livingstone. Price 10s. 6d. net.)

THE new edition of this well-known and valuable manual will be welcomed by the numerous candidates for examination whose subjects are embraced within its compass. It is admirably adapted for its purpose of affording the student concise information on hygiene, sanitation, heat, water, food, and clothing, ventilation, drainage, climate, zymotic diseases, disinfection, and kindred subjects Two new chapters on vital statistics and on eugenics and bionomics have been added. These contain in a necessarily condensed form much valuable matter directed to the business of helping the student to think, but the last chapter is far too brief for the far-reaching conclusions enunciated.

**Military Sanitation: a Handbook for Soldiers.** By COLONEL ROBERT J. BLACKHAM, C.B., C.M.G., C.I.E., D.S.O., &c. Third edition. (London: John Bale, Sons, and Danielsson, Ltd. Pp. 136. Price 10s. 6d.)

THE Army is to be congratulated on the publication of a third edition of Colonel Blackham's excellent aid to the maintenance of the health and strength of the fighting forces. A great deal of the experience of the recent war, more especially on the European fronts, has been embodied, and the style is so lucid and simple that the officers of the fighting branches should have no difficulty in understanding the technical descriptions.

The subjects are well arranged under such chapters as Air, Water, Food, Clothing, Camps, Marching, Special War Diseases, and Venereal Diseases.

There is much that is valuable in each, but no guide to army sanitation in the field is complete without reference to the "Sanitary Sections," which contributed so largely to the reduction of sick wastage in the late war. It is to the amplified Sanitary Section of the future—the "Sanitary Battalion" or "Sanitary Corps"—that we look for the further reduction of wastage. The doctors having successfully fought enteric, dysentery, tetanus, cerebro-spinal fever, wound sepsis, etc., etc., it is incumbent on the "Sanitary Corps" to attain equal success in the future.

The duties of "Sanitary Sections" are so manifold and their relation to the various branches of the Army so intricate, that a great deal of education of all ranks is required in order to enlist their co-operation and goodwill.

**The Essentials of Tropical Medicine.** By DR. W. E. MASTERMAN, M.D., M.R.C.S., L.R.C.P. (John Bale, Sons & Danielsson, Ltd., Oxford House. 1920.)

THE book is intended as a digest or compendium of tropical medicine. The subject has grown so of late that some cutting down into more reasonable compass is certainly required, and this Dr. Masterman has attempted. It is a difficult task to please everyone, and there are points in the work which the keen-eyed critic will quickly fall upon and rend to pieces. On the whole, however, the book contains quite a large amount of accurate information, well put together, and easily assimilable by the ordinary individual. It is quite well illustrated, also, and this should help the explanation of the different diseases and impress the facts upon the memory of the reader. The student might well have a run through its pages prior to his examinations, and the practitioner abroad will also find it useful.

